# Lynch syndrome-associated lung cancer: pitfalls of an immunotherapy-based treatment strategy in an unusual tumor type

**DOI:** 10.37349/etat.2021.00044

**Published:** 2021-06-28

**Authors:** Elena Maccaroni, Edoardo Lenci, Veronica Agostinelli, Valeria Cognigni, Riccardo Giampieri, Paola Mazzanti, Marzia Di Pietro Paolo, Francesca Bianchi, Cristiana Brugiati, Laura Belvederesi, Silvia Pagliaretta, Alessandra Mandolesi, Marina Scarpelli, Alberto Murrone, Francesca Morgese, Zelmira Ballatore, Rossana Berardi

**Affiliations:** 1 Clinica Oncologica, Azienda Ospedaliero Universitaria Ospedali Riuniti Umberto I G M Lancisi G Salesi, 60126 Ancona, Italy; 2 Medical Oncology Unit, Università Politecnica delle Marche, 60126 Ancona, Italy; 3 SOD Anatomia Patologica, Azienda Ospedaliero Universitaria Ospedali Riuniti Università Politecnica delle Marche, 60126 Ancona, Italy; University of Southampton, UK

**Keywords:** Lynch syndrome, immunotherapy, non-small cell lung cancer, microsatellite instability-high, pembrolizumab

## Abstract

Lynch syndrome is a hereditary cancer predisposition syndrome caused by germline alterations in mismatch repair (MMR) genes leading to increased risk of colon cancer as well as other cancer types. Non-small cell lung cancer (NSCLC) is not among typical Lynch syndrome-associated tumors: pembrolizumab, an immune checkpoint inhibitor, is actually approved for the treatment of NSCLC patients and represents a promising treatment option for patients with advanced metastatic MMR-deficient cancer, regardless of tumor origin. This case report describes the clinical presentation and management of a 74-year-old female with a history of rectal adenocarcinoma and ovarian cancer, who has a documented frameshift pathogenic variant in the exon 8 of *MSH6* gene and an intronic variant in the *BRCA2* gene (classified as a variant of uncertain significance), affected by NSCLC with brain metastases. Despite these premises, the patient was treated with pembrolizumab and she did not benefit from this kind of treatment.

## Introduction

Lynch syndrome, formerly known as hereditary non-polyposis colorectal cancer (CRC), is an autosomal dominant genetic disorder that leads to the development of CRCs and several other tumors, including other gastro-intestinal cancer, endometrium, ovary, upper urinary tract, prostate, brain, and some different types of skin cancers [1, 2]. Moreover, Lynch syndrome-associated CRC might have a better prognosis compared to sporadic CRC [3, 4]. Lynch syndrome is caused by germline pathogenic variants in some of the genes involved in DNA replication errors repair through mismatch repair (MMR) [MutL homolog 1 (*MLH1*), MutS homolog (*MSH*)*2*, *MSH6*, and PMS1 Homolog 2 (*PMS2*)] system, as well as germline deletion of the gene *EPCAM*, which is not an MMR gene but causes inactivation of the *MSH2* gene with the methylation of its promoter. These two forms of mutations do account for almost 90% of identified defects [5].

Failure of DNA MMR leads to a phenomenon defined as microsatellite instability (MSI), characterized by change of the length of simple, repetitive nucleotide sequences that occur throughout the genome and to higher tumor mutational load. MSI represents a hallmark of this syndrome [6]. Moreover, Lynch syndrome-associated CRC might have a better prognosis compared to sporadic CRC [5, 6].

Recently, immune checkpoint inhibitor (ICI) has been tested in clinical trials in patients with MMR-deficient (dMMR) CRC and non-CRC pre-treated patients, with promising results. In a phase II trial, anti-programmed cell death protein 1 (PD-1) agents achieved 71% overall response rate in patients with solid tumors excluding CRC with MSI-high (MSI-H) [7]. In another study, 86 patients with CRC or non-CRC with MSI-H were treated with anti-programmed cell death ligand 1 (PD-L1) antibodies and the objective response rate was 53%. To note, in this study, 45% of patients had a detected germline mutation in the MMR genes or a known Lynch syndrome [8]. It has been hypothesized that cancers associated with MSI-H phenotype show a higher mutation-associated neoantigens’ expression and this could explain the increased response to immunotherapy [8]. Indeed, the use of immunotherapy could improve outcomes in patients with MSI-H cancer, including ones affected by Lynch syndrome [9].

On top of that, at the last American Society of Clinical Oncology (ASCO) meeting, in a phase III trial (KEYNOTE-177) performed on patients with MSI-H metastatic CRC, immunotherapy with pembrolizumab was proven to be more effective than chemotherapy based on the best choice of investigator: both median progression-free survival [mPFS, 16.5 *vs.* 8.2 months, hazard ratio (HR): 0.60, 95% CI: 0.45–0.80, *P* = 0.0002] and overall response rate (43.8% *vs.* 33.1%, *P* = 0.0275) were significantly better with immunotherapy *vs.* standard 1st line chemotherapy options [10].

Immunotherapy (anti-PD-1 or anti-PD-L1 agents) represents a standard of care in non-small cell lung cancer (NSCLC) patients, even when clinical presentations have complex management such as those with brain metastases [11]. Clearly, both multidisciplinary evaluation and treatment are mandatory for every single case, but it can be safely assumed that immunotherapy can play a role in the management of brain metastases, too [12]. In a phase II trial, 18 patients with NSCLC and brain metastases were treated with pembrolizumab: six patients showed a partial response and there was high concordance when comparing the outcome of systemic and brain metastases response [13]. In a pooled analysis of four clinical trials, pembrolizumab in first-line setting showed improved clinical benefit when compared with chemotherapy and provided similar outcomes irrespectively from the presence of brain metastases. Patients treated with pembrolizumab had a significant reduction of death risk compared to patients treated with chemotherapy, even if brain metastases were present, and independently from PD-L1 expression (HR: 0.78 in NSCLC with PD-L1 ≥ 50%; HR: 0.83 in NSCLC with PD-L1 ≥ 1%) [12].

At present, in the literature, there are only a few cases of patients with Lynch syndrome who also received a diagnosis of metastatic NSCLC and treated with ICI [14–16].

Therefore, we report the case of a Lynch syndrome carrier affected by NSCLC with brain metastases, treated with pembrolizumab, with a history of previous ovarian and rectal cancer.

## Case report

A 74-year-old never-smoker woman has been referred to our center (Medical Oncology Clinics, University Hospital Ancona) in 1987. Her main comorbidities included hypertension, hypercholesterolemia, type II diabetes, and chronic kidney disease. The patient developed ovarian cancer in 1987 at the age of 43 and underwent radical surgery and subsequent adjuvant chemotherapy with cisplatin, doxorubicin, and cyclophosphamide. Unfortunately, detailed information regarding tumor histology, grading, and staging is not available nowadays.

In 2005, at the age of 61, she also developed a rectal adenocarcinoma, treated with surgical resection and adjuvant chemotherapy with fluorouracil and folinic acid (FUFA regimen).

Her third cancer occurred in May 2016, at the age of 72, when she developed lung cancer in the right lower lobe, treated with right lung lobectomy; the histological report showed a moderately differentiated lung adenocarcinoma, pathologic stage IA (pT1b N0 M0).

Molecular characterization on primary lung tumor was negative for any mutation of *EGFR*, *KRAS*, *NRAS*, *BRAF*, *PIK3CA*, *ERBB2*, *DDR2*, *MAP2K1*, *RET* genes, and also negative for *ALK*-translocation. Molecular characterization was performed with Sequenom matrix-assisted laser desorption/ionization time-of-flight mass spectrometry (MALDI-TOF) technology using Myriapod Lung Status Kit ^®^ (Diatech Pharmacogenetics) [17]. Also PD-L1 expression (PD-L1 < 1%; PD-L1 evaluated with Dako platform, 22C3 pharmDx ^TM^) resulted negative.

Due to the occurrence of multiple tumors, after surgical lung resection, genetic counseling was offered to investigate, for the first time, her cancer susceptibility.

**Figure 1.**
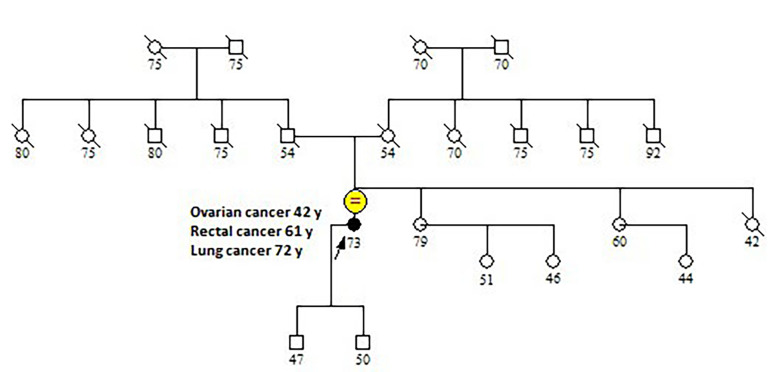
Family pedigree. The arrow indicates the proband. The patient did not have any family history of Lynch syndrome-related tumors

Detailed patient family history was collected: pedigree was drawn until the fourth generation. All relatives’ data collected included sex, tumor size, age at diagnosis together with current age or age of death. She had no other cancer cases until her second and third-degree family members (Figure 1), even if she had only a little information’s regarding her maternal and paternal grandparents. She accepted to be screened for germline pathogenic variants in *MLH1*, *MSH2*, *MSH6* genes, largely known as causative of Lynch syndrome, and also in *BRCA1* and *BRCA2* genes, involved in hereditary breast and ovarian cancer syndrome.

Mutation analysis was performed on genomic DNA, isolated from peripheral blood lymphocytes, according to standard procedures.

Both PCR and direct sequencing (Sanger’s method) and multiplex ligation-dependent probe amplification analysis were used to study the entire *MLH1*, *MSH2*, *MSH6*, *BRCA1*, and *BRCA2* genes coding sequence, including flanking intronic regions.

The genetic testing results showed a frameshift pathogenic variant in the exon 8 of *MSH6* gene, c.3742_3745del, p.(His1248Thrfs*4), that consists in deletion of four nucleotides (CACT) in 3,742 positions, thus causing a replacement of histidine with threonine and, subsequently, the insertion of a stop codon in the corresponding encoded protein, that results incomplete and inactive.

On top of that, we also detected an intronic variant in the *BRCA2* gene [intron 9, c.793+46_793+48del, p.(?)] classified as variants of uncertain significance (VUS) [18].

After the diagnosis of Lynch syndrome, the patient continued her follow-up, but in January 2018, a positron emission tomography (PET)/computed tomography (CT) scan with fluorine-18 (^18^F) fluorodeoxyglucose tracer showed an area of increased uptake in the upper left lobe, suspicious for lung metastasis, and other two areas of increased uptake in right paratracheal lymph nodes and the bare spaces. Endobronchial ultrasound-guided transbronchial needle aspiration (EBUS-TBNA) confirmed nodal disease recurrence of lung cancer.

At relapse, the patient was 74 years old and had a performance status of 1 according to eastern cooperative oncology group (ECOG) classification, so she was treated with stereotactic radiotherapy: 40 Gy in 5 fractions were administered on mediastinal nodal relapse and 39 Gy were administered on left lung metastasis. In November 2018, however, a chest and abdomen CT scan revealed a relevant progressive disease with multiple bilateral lung metastases.

The patient still had ECOG 1 performance status, and, due to concomitant comorbidities, she was treated with a systemic monotherapy with carboplatin area under the curve (AUC) 4.

In January 2019, three weeks after the first carboplatin course, the patient had a severe headache associated with neck stiffness, so she underwent a cranial CT scan, which revealed a huge intraparenchymal hemorrhagic lesion in the right frontal lobe. She was admitted to the Neurosurgery Clinic where she underwent an urgent surgical intervention, with the removal of a bleeding mass in the right frontal lobe.

**Figure 2.**
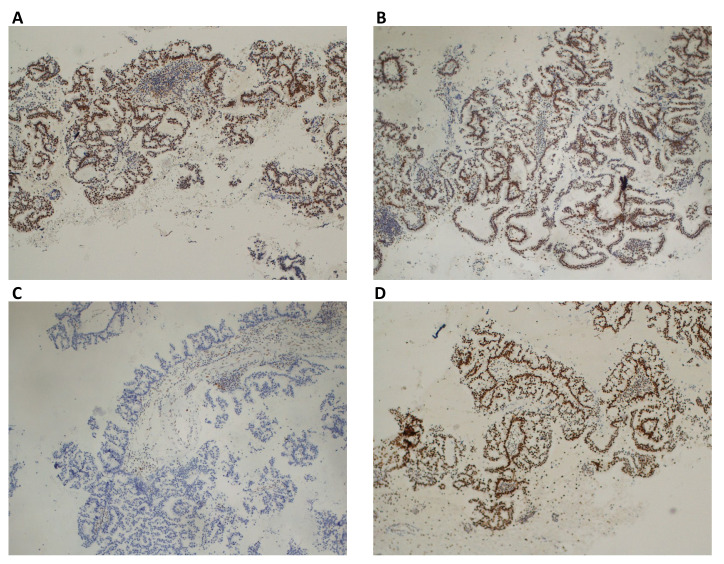
Immunohistochemical staining of MMR proteins evaluated on lung cancer. (A) Normal nuclear expression of MLH1protein; (B) normal nuclear expression of MSH2 protein; (C) lack of expression of MSH6 protein; (D) normal nuclear expression of PMS2 protein. Magnification ×100

The histopathological report of the brain mass confirmed the presence of a lung adenocarcinoma metastasis. Considering the status of Lynch syndrome carrier, we also assessed MLH1, MSH2, MSH6, and PMS2 by immunohistochemistry (IHC) on tumor tissue, and we found that the expression of MSH6 was lost (Figure 2). Primary antibodies used for IHC were: anti-MLH1 (clone ES05), anti-MSH2 (clone FE11), anti-MSH6 (clone EP49), anti-PMS2 (clone EP49) (all from Dako). Also, MSI status was evaluated: genomic DNA was isolated from tumor samples and corresponding normal tissues as previously described [19, 20]. Tumors were examined for MSI using the 5-marker panel (two mononucleotide repeats—BAT25 and BAT26 and three dinucleotide repeats—D2S123, D5S346, and D17S250) recommended by the National Cancer Institute Workshop on MSI for Cancer Detection and Familial Predisposition. Tumors were classified as highly unstable (MSI-H) if at least 40% of the markers showed instability [4]. MSI-status showed MSI-H: two out of the five loci studied (Bethesda panel) resulted in altered (40% MSI), BAT25, and BAT26.

The post-surgery recovery was complicated by cardiogenic shock due to torsade de pointes and severe acute renal failure, which required a long hospitalization.

After these critical events, the patient’s clinical conditions worsened, ECOG 2–3, and consequently, she was deemed to be no longer suitable for further anti-cancer treatment and addressed to best supportive care.

Surprisingly, a few months later, in May 2019, the patient’s conditions improved (ECOG 1), but a chest/abdomen CT scan showed progressive disease with an increase in number and size of lung metastases, while a brain magnetic resonance imaging was negative for secondary lesions. Carcinoembryonic antigen (CEA) plasmatic level was high [CEA: 14 ng/mL; normal value (NV) < 5 ng/mL].

Therefore, due to the improvement of patient performance status and progressive disease, treatment with pembrolizumab was prescribed. However, at that time, in our country (Italy), treatment with this drug was not approved due to the lack of expression of PD-L1 in the primary tumor.

Based on the fact that PD-L1 expression heterogeneity has been demonstrated to be present in lung cancer [19, 21, 22], we requested second molecular testing on lymph node biopsy that showed weak PD-L1 positivity (PD-L1 = 5%). We started pembrolizumab flat dose 200 mg every 3 weeks, which was approved as a second-line treatment of patients with NSCLC and PD-L1 > 1% in Italy. This treatment resulted in a further improvement of clinical conditions and her CEA plasmatic level decreased (in August CEA: 6.2 ng/mL).

After 4 months of treatment, in September 2019, a CT scan and brain magnetic resonance imaging showed disease stability.

Unfortunately, in November 2019, the patient was urgently admitted to our center due to the onset of severe neurological symptoms and seizures. Brain CT scan confirmed massive disease progression in the brain. The clinical condition rapidly deteriorated and the patient was addressed to palliative care. The patient died in December 2019.

**Figure 3.**
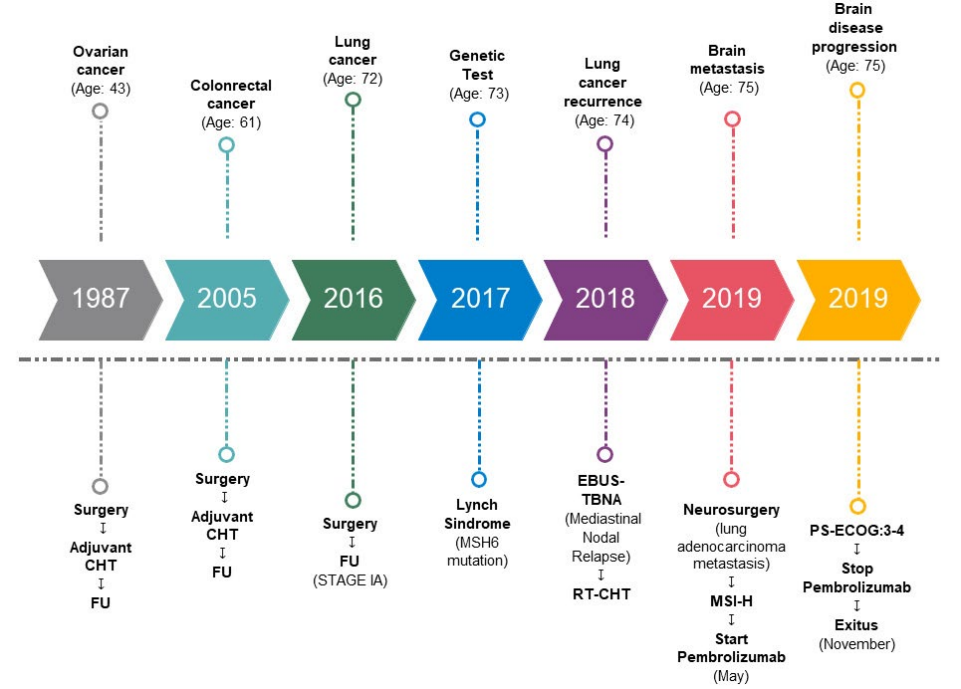
Timeline events. CHT: chemotherapy; FU: follow up; RT: radiotherapy; PS-ECOG: performance status-ECOG

Timeline events are briefly summarized in Figure 3.

## Discussion

Lynch syndrome carriers harbor germline loss-of-function variants in MMR genes (*MLH1*, *MSH2*, *MSH6*, and *PMS2*) and have an increased risk of specific cancer types, particularly CRC, endometrial, ovarian, and gastric carcinomas [1, 2].

Data regarding Lynch syndrome and lung cancer risk are scarce, but a pan-cancer study published in 2016 reported that germline *MSH6* pathogenic variants were present in about 1% (2/191) of lung cancer patients [20]. As lung cancer is not one of the recognized Lynch syndrome-associated tumors, the relationship between lung cancer risk and germline MMR mutations requires further investigations.

Recent work by Sun et al. [23] was performed to investigate whether lung cancers occurring in Lynch syndrome carriers were due to MSI-H status or were instead sporadic cancers whose driver was different from the increased mutational load associated with MSI-H status. Pathogenic or likely pathogenic germline mutations in *PMS2*, *MSH2*, or *MSH6* were retrospectively detected in 0.5% (6/1179) of lung cancer patients, but none of these six patients exhibited MSI or loss of MMR protein expression, suggesting that lung cancers in these six Lynch syndrome patients developed sporadically and were unrelated to the underlying Lynch syndrome diagnosis [23]. The authors concluded that, without proper family history reconstruction and genetic counseling, the probability of identifying Lynch syndrome associated with lung cancer is particularly low (0.5%), thus supporting the “rarity” of our case report. On top of that, the authors of this study suggested that, even if an underlying Lynch syndrome might be present, the occurrence of lung cancer is mainly due to sporadic mutations, unrelated to the altered DNA repair mechanism. This fact alone might explain why, in our case, even though Lynch syndrome was detected and *MSH6* lack was proven, differently from what one could expect, pembrolizumab treatment did not work as well as expected [23].

Conversely, in our patient, lung adenocarcinoma immunohistochemical staining showed loss of MSH6 expression both in primary tumor and in brain metastasis, suggesting that its development could be traced back to MSI-H status. Furthermore, no other relevant mutations (such as *EGFR*, *ROS1*, *KRAS*, *ALK*) were found, thus further suggesting that one of the “drivers” of lung cancer development, in this case, might be also related to the underlying Lynch syndrome.

In this case report, we treated the patient with Lynch syndrome-related NSCLC with brain metastases with pembrolizumab. DMMR and MSI-H tumors show higher expression of mutation-associated neoantigens and it could be a possible explanation of increased response to immunotherapy.

Despite expectations, our patient did not respond to pembrolizumab, but only had disease stabilization for a few months.

Because only a subset of NSCLC patients will respond to immunotherapy, the identification of biomarkers that predict benefit from PD-1 inhibitors is now of great interest. PD-L1 represents one of the most important predictive factors in lung cancer patients treated with ICI, and our case suggests that for NSCLC patients, MSI-H/dMMR status might not be as relevant as PD-L1 expression (or lack thereof) as a predictor of efficacy to ICIs. In our patient, both the very low PD-L1 expression and the poor clinical condition could explain the scarce response to pembrolizumab.

One finding suggests that rare phenotypes of cancers such as lung adenocarcinoma may occur in Lynch syndrome carriers, but factors influencing prognosis and response to treatment, such as chemotherapy and checkpoint inhibitors, are still unknown and requires further investigations.

## Abbreviations

CEA: carcinoembryonic antigen
CRC: colorectal cancer
CT: computed tomography
dMMR: mismatch repair-deficient
ECOG: eastern cooperative oncology group
HR: hazard ratio
ICI: immune checkpoint inhibitor
MLH1: MutL homolog 1
MMR: mismatch repair
MSH: MutS homolog
MSI: microsatellite instability
MSI-H: microsatellite instability-high
NSCLC: non-small cell lung cancer
PD-1: programmed cell death protein 1
PD-L1: programmed cell death ligand 1
PMS2: PMS1 Homolog 2

## Declarations

### Copyright

© The Author(s) 2021.
